# Effects of the blood urea nitrogen to creatinine ratio on haemorrhagic transformation in AIS patients with diabetes mellitus

**DOI:** 10.1186/s12883-019-1290-x

**Published:** 2019-04-13

**Authors:** Linghui Deng, Shi Qiu, Changyi Wang, Haiyang Bian, Lu Wang, Yuxiao Li, Bo Wu, Ming Liu

**Affiliations:** 1 Center of Cerebrovascular Diseases, Department of Neurology, West China Hospital, Sichuan University, Chengdu, Sichuan China; 2 Department of Urology, Institute of Urology, West China Hospital, Sichuan University, Chengdu, Sichuan China; 3 Center of Biomedical big data, West China Hospital, Sichuan University, Chengdu, Sichuan China; 4 Department of Epidemiology and Biostatistics, School of Public Health, Peking University, Beijing, China

**Keywords:** Blood urea nitrogen, Creatinine, Ischaemic stroke, Haemorrhagic transformation, Nonlinear relationship

## Abstract

**Background:**

The effect of the blood urea nitrogen (BUN) to creatinine (Cr) ratio (henceforth BUN/Cr) on haemorrhagic transformation (HT) of acute ischaemic stroke (AIS) patients is unclear.

**Methods:**

AIS patients in the West China Hospital, Sichuan University, Chengdu, China, admitted within seven days from stroke onset (2012–2016) were included in the study. Baseline data, including BUN and Cr levels, were collected. The outcome was defined as HT during hospitalization.

**Results:**

In this study, 1738 participants with an average age of 62.7 ± 14.0 years were included. After adjusting potential confounders (age, blood platelet, albumin, stroke severity, triglycerides and low-density lipoprotein [LDL]), multivariate logistic regression analyses indicated that BUN/Cr is independently associated with HT. The nonlinear relation between BUN/Cr and HT was explored in a dose-dependent manner, with an apparent inflection point of 30.71. On the left and right sides of the inflection point, the odds ratio (OR) and 95% confidence interval (CI) were 1.05 (1.02–1.08) and 0.96 (0.88–1.05), respectively. Interaction between BUN/Cr and diabetes mellitus (DM) and HT (*P* for interaction = 0.0395) was noted. BUN/Cr showed positive correlation with HT in DM patients (OR = 1.07; 95% CI: [1.02, 1.12]) but no significant relationship with HT in patients without DM.

**Conclusion:**

BUN/Cr is significantly associated with HT in AIS patients in a linear fashion, with an apparent cut point demarcating the HT difference. When the patients have DM, BUN/Cr is positively correlated with HT. These results support a revision in how we anticipate the prognosis for AIS patients.

**Electronic supplementary material:**

The online version of this article (10.1186/s12883-019-1290-x) contains supplementary material, which is available to authorized users.

## Background

Acute ischaemic stroke (AIS) is a devastating condition with high mortality and morbidity, which is often complicated by haemorrhagic transformation (HT) and is potentially linked to clinical deterioration [1, 2]. The active exploration of the risk factors for HT has clinical significance as it could help clinicians identify potential risks, adjust the therapeutic schedule, reduce the occurrence of HT and consequently improve the quality of life for patients. Approximately 30% of AIS patients present with renal dysfunction [3], which is considered an independent prognostic indicator of poor clinical outcomes [4]. Exploring biomarkers of kidney impairment could be helpful to evaluating cerebral microvascular risk and correlation of stroke complications [5]. Studies have demonstrated that the estimated glomerular filtration rate (eGFR) is associated with HT [6, 7]. Besides the eGFR, blood urea nitrogen (BUN) and creatinine (Cr) are also used for evaluating renal function.

Recently, the BUN-to-Cr ratio (BUN/Cr) has emerged as an independent prognostic indicator of poor outcomes in different disease conditions, such as acute and chronic heart failure [8–11], acute and chronic kidney injury [12, 13] and ischaemic stroke [14]. Studies have indicated that an elevated BUN/Cr may be a potential marker for early neurological deterioration and a three-month outcome in AIS patients [14–18]. In addition, an elevated BUN/Cr is reportedly an independent risk factor for venous thromboembolism in AIS patients [19]. However, there was no research focusing on the association between BUN/Cr and the risk of HT in AIS. Therefore, in this study, we investigated the association between BUN/Cr and HT in AIS patients.

## Methods

### Study population

In this study, we included adult patients admitted to West China Hospital, Sichuan University, Chengdu, China, within 7 days of first-ever AIS onset between January 2012 and December 2016. Given the retrospective nature of the study, requirement for informed consent was waived by the Institution review board of of West China Hospital, Sichuan University, Chengdu, People’s Republic of China. All patients were diagnosed with AIS on the basis of the World Health Organization criteria and the *Trial of Org 10,172 in Acute Stroke Treatment* criteria [20, 21]. The diagnosis was further confirmed by magnetic resonance imaging (MRI) or computed tomography (CT). The patients were entered consecutively and prospectively into the Chengdu Stroke Registry [22]. Informed consent of the patients was not needed, because the current study was a database-based analysis not experimental research on humans. Patients were, however, excluded from the study if: (i) they were diagnosed with primary subarachnoid haemorrhage or intracerebral haemorrhage on the basis of first-time head CT scans (ii) they had severe liver disease or end-stage renal disease or (iii) their BUN or Cr values were unavailable on admission.

### Data collection and outcome

The following data were collected by reviewing the patients’ medical records: demographic characteristics (age and gender), stroke risk factors (history of diabetes mellitus [DM], hypertension, dyslipidaemia, atrial fibrillation [AF], smoking status, alcohol consumption), interval of symptom onset, stroke severity on admission and laboratory data. In addition, the data on stroke severity was assessed using the National Institutes of Health Stroke Scale (NIHSS) from the case files [23]. HT was defined as haemorrhage inside the infarct region or parenchyma outside the infarct territory on a follow-up CT or MRI [24].

### Statistical analysis

In this study, data were presented as mean ± standard deviation (normal distribution) or median (quartile; skewed distribution) for continuous variables and as a frequency or a percentage for categorical variables. The statistical difference between within-population characteristics were determined using analysis of variance (normal distribution), Kruskal–Wallis *H* test (skewed distribution) and chi-squared test (categorical variables). The statistical software packages used were R (http://www.R-project.org, The R Foundation) and EmpowerStats (http://www.empowerstats.com, X&Y Solutions, Inc., Boston, MA, USA). First, we used a univariate linear regression model to assess the relationship between BUN/Cr and HT. Both univariate and multivariate models with crude and adjusted odds ratios (ORs) were listed. We used the generalized additive model (GAM) model to adjust the continuous variables in the model II. Covariates significantly associated with the response variable (*P* < 0.05) or changed the effect estimate by 10% or more were retained in the final adjusted model [25]. Second, we used the GAM to identify the nonlinear relationship between BUN/Cr and HT. If a nonlinear correlation was detected, a two-piecewise linear regression model was then used to determine the threshold effect of BUN/Cr on HT in accordance with the smoothing plot. When the BUN/Cr:HT ratio appeared obvious in the smoothing plot, the inflection point was figured automatically by the recursive method using the maximum model likelihood [26]. Finally, we inspected the modification and interaction of subgroups using the likelihood ratio test. Two-tailed *P* < 0.05 was considered statistically significant.

## Results

### Study participants and baseline characteristics

Of the 3458 participants in the Chengdu Stroke Registry, our data analyses were limited to 1738 subjects from West China Hospital. Please refer to Additional file 1: Figure S1 for a flowchart. The average age of the participants was 62.7 ± 14.0 years, and ~ 60.2% of them were male. The average baseline NIHSS was 6.53 ± 6.26. Table 1 lists the baseline characteristics. Compared to the high level (T3) of BUN/Cr group, participants in the other two groups (T1 and T2) were younger and had a higher percentage of males, higher baseline Cr and higher alcohol intake; were smokers; had a lower baseline NIHSS, BUN and high-density lipoprotein (HDL); and had a lower rate of history of AF and severe stroke.

**Table 1:** Baseline Characteristics of participants

BUN/Cr	Tertile 1	Tertile 2	Tertile 3	*P*-value
N	578	580	580	
Age (years, mean ± sd)	59.96 ± 14.56	63.24 ± 13.96	64.92 ± 13.06	< 0.001*
Male,n (%)	438 (75.78%)	369 (63.62%)	240 (41.38%)	< 0.001*
Intervals between symptoms onset to admission (minutes, mean ± sd)	60.90 ± 47.73	60.05 ± 48.94	62.87 ± 51.11	0.61
Baseline NIHSS score (mean ± sd)	5.65 ± 5.80	6.46 ± 6.20	6.90 ± 6.58	< 0.01*
Blood platelet (*10^9^/L, mean ± sd)	173.33 ± 69.14	165.83 ± 60.51	168.80 ± 64.37	0.14
Albumin(g/L, mean ± sd)	40.84 ± 4.53	41.00 ± 4.48	40.45 ± 4.66	0.11
BUN (mg/dL, mean ± sd)	13.30 ± 6.80	16.17 ± 4.76	20.70 ± 6.90	< 0.001*
Cr (mg/dL, mean ± sd)	1.09 ± 0.70	0.92 ± 0.27	0.81 ± 0.27	< 0.001*
Triglyceride (mmol/L, mean ± sd)	1.64 ± 1.16	1.60 ± 1.06	1.55 ± 1.12	0.38
Total cholesterol (mmol/L, mean ± sd)	4.41 ± 1.11	4.44 ± 1.13	4.43 ± 1.10	0.90
HDL (mmol/L, mean ± sd)	1.27 ± 0.38	1.29 ± 0.38	1.32 ± 0.41	< 0.05*
LDL (mmol/L, mean ± sd)	2.60 ± 0.95	2.66 ± 0.97	2.59 ± 0.93	0.46
Hypertension, n (%)	278 (48.10%)	296 (51.03%)	290 (50.00%)	0.60
Diabete Mellitus, n (%)	89 (15.40%)	112 (19.31%)	119 (20.52%)	0.06
Hyperlipidemia, n (%)	30 (5.19%)	27 (4.66%)	27 (4.66%)	0.89
Atrial Fibrillation, n (%)	25 (4.33%)	45 (7.76%)	64 (11.03%)	< 0.001*
Alcohol intake, n (%)	185 (32.01%)	156 (26.90%)	104 (17.93%)	< 0.001*
Current smoking, n (%)	249 (43.08%)	211 (36.38%)	145 (25.00%)	< 0.001*
Stroke severity				< 0.01*
Baseline NIHSS score < 15, n (%)	530 (91.70%)	519 (89.48%)	499 (86.03%)	
Baseline NIHSS score > =15, n (%)	48 (8.30%)	61 (10.52%)	81 (13.97%)	

* Represents the unit of blood platelet e.g., the normal range of blood platelet is 4-10*10^9^/L

*BUN* Blood urea nitrogen, *Cr* Creatinine, *NIHSS* National Institutes of Health Stroke scale, *HDL* High-density lipoprotein, *LDL* Low-density lipoprotein

### Univariate analysis

A univariate linear regression model was used to evaluate the association between BUN/Cr and HT. Additional file 1: Table S1 summarizes the results of the analysis. The analysis revealed that age, a history of AF and stroke severity are correlated with a higher risk of HT, whereas baseline blood platelet, albumin, cholesterol and low-density lipoprotein (LDL) levels are associated with a lower risk of HT (Additional file 1: Table S1).

### Results of analysis of the BUN/Cr–HT relationship

As shown in Table 2, BUN/Cr displayed a positive correlation with HT in the crude model (OR = 1.03; 95% CI: [1.01–1.05]; *P* = 0.0019). In the adjusted model (age, blood platelet, albumin, baseline NIHSS, triglycerides and LDL), the result remained stable (OR = 1.02; 95% CI: [1.00–1.05]; *P* = 0.03). For sensitivity analysis, BUN/Cr was considered a categorical variable (tripartite) and a similar trend was found (*P* for trend < 0.01; see Table 2).

**Table 2:** Relationship between BUN/Cr and HT in different models

Variable	Crude model (β, 95%CI, *P*)	Model I (β, 95%CI, *P*)	Model II (β, 95%CI, *P*)
BUN/Cr	1.03 (1.01, 1.05) < 0.01	1.03 (1.00, 1.05) 0.02	1.02 (1.00, 1.05) 0.03
BUN/Cr (tertile)			
T1	Ref	Ref	Ref
T2	0.97 (0.61, 1.53) 0.89	0.89 (0.56, 1.42) 0.63	0.89 (0.56, 1.42) 0.63
T3	1.88 (1.25, 2.82) < 0.01	1.63 (1.08, 2.47) 0.02	1.62 (1.07, 2.45) 0.02

*p* for trend, < 0.01, < 0.01, < 0.01

Crude model: we did not adjust other covariants

Model I: we adjusted Age; Blood platelet; Albumin; Stroke severity; Triglyceride; LDL

Model II: we adjusted Age (Smooth); Blood platelet (Smooth); Albumin (Smooth); Stroke severity; Triglyceride (Smooth); LDL (Smooth).LDL = low-density lipoprotein

*CI* Confidence interval, *Ref* Reference, *BUN* Blood urea nitrogen, *Cr* Creatinine

### Analyses of the BUN/Cr–HT nonlinear relationship

**Fig. 1:**
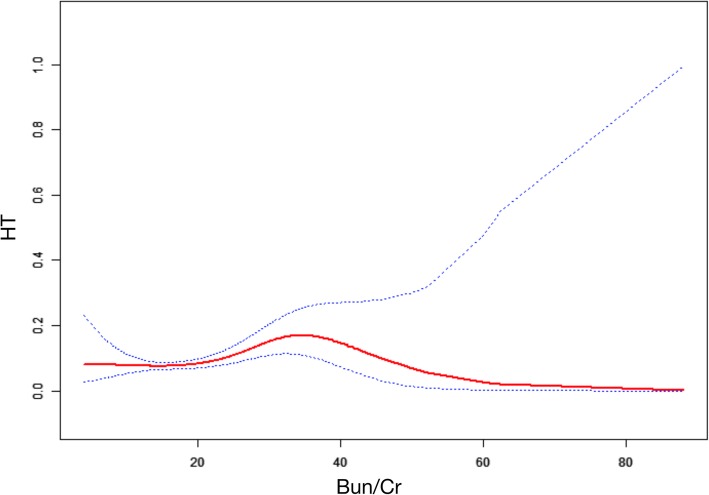
The non-linear relationship between Bun/Cr and HT

It is essential to analyse nonlinear relationships for continuous variables. In this study (Fig. 1), we detected a nonlinear relationship between BUN/Cr and HT after adjusting the age, blood platelet, albumin, stroke severity, triglycerides and LDL. Using the two-piecewise linear regression model, we calculated the inflection point as 30.71. The OR (95% CI) and *P* values were 1.05 (1.02, 1.08) and 0.0029, respectively, on the left of the inflection point. However, on the right of the inflection point, the BUN/Cr–HT relationship displayed insignificant values: OR = 0.96; 95% CI: (0.88, 1.05); *P* = 0.36. See Fig. 1 and Table 3.

**Table 3:** The results of two-piecewise linear regression model

Inflection point of BUN/Cr	Effect size (95%CI)	*P* value
< 30.71	1.05 (1.02, 1.08)	< 0.01
> 30.71	0.96 (0.88, 1.05)	0.3629

Low-density lipoprotein, *BUN* Blood urea nitrogen, *Cr* Creatinine

Adjust: Age; Blood platelet; Albumin; Stroke severity; Triglyceride; LDL. *LDL*

### Subgroup analyses

The results of our subgroup analyses are presented in Table 4. After adjusting potential confounders, we found that the test for interaction was statistically significant for a history of DM (*P* for interaction = 0.04) but not for age, sex, history of hypertension, hyperlipidaemia, AF, blood platelet, albumin, stroke severity, triglycerides, total cholesterol, HDL and LDL. We also found evidence of BUN/Cr–DM interaction. The effect of BUN/Cr on HT significantly differed between patients with and without DM. BUN/Cr had a positive correlation with HT (OR = 1.07, 95% CI: [1.02, 1.12]) in DM patients but no significant relationship with HT in patients without DM. Moreover, we observed that BUN/Cr was positively correlated with HT when BUN/Cr was less than 30.71 (Table 4).

**Table 4:** Effect size of BUN/Cr on HT in prespecified and exploratory subgroups

Characteristic	No of participants	Effect size(95%CI), *p* value	*P* for interaction
BUN/Cr			0.02*
< 30.71	1644	1.04 (1.00, 1.07), 0.04	
30.71	89	0.91 (0.80, 1.03), 0.15	
Age (year)			0.22
< 65	910	1.01 (0.98, 1.05), 0.51	
≧65	823	1.04 (1.01, 1.07), 0.01	
Sex			0.89
Female	689	1.02 (0.99, 1.05), 0.11	
Male	1044	1.03 (0.99, 1.07), 0.19	
Hypertention			0.49
No	873	1.03 (1.00, 1.06), 0.03	
Yes	860	1.02 (0.98, 1.05), 0.41	
Diabetes Mellitus			0.04*
No	1415	1.01 (0.99, 1.04), 0.39	
Yes	318	1.07 (1.02, 1.12), < 0.01	
Hyperlipidemia			0.15
No	1651	1.03 (1.01, 1.05), 0.02	
Yes	82	0.87 (0.68, 1.12), 0.29	
Atrial Fibrillation			0.86
No	1599	1.02 (1.00, 1.05), 0.05	
Yes	134	1.03 (0.96, 1.10), 0.40	
Alcohol intake			0.32
No	1288	1.02 (1.00, 1.05), 0.09	
Yes	445	1.05 (0.99, 1.12), 0.07	
Current smoking			0.77
No	1129	1.02 (1.00, 1.05), 0.07	
Yes	604	1.03 (0.98, 1.08), 0.19	
Stroke severity			0.48
No	1543	1.03 (1.01, 1.05), 0.02	
Yes	190	1.01 (0.96, 1.06), 0.69	
Blood platelet			0.49
No	864	1.03 (1.00, 1.06), 0.03	
Yes	869	1.02 (0.98, 1.05), 0.30	
Albumin			0.31
No	861	1.02 (0.99,1.04), 0.29	
Yes	873	1.04 (1.00, 1.08), 0.03	
Triglyceride			0.24
Low	867	1.01 (0.99, 1.04), 0.33	
High	866	1.04 (1.01, 1.08), 0.02	
Total cholesterol			0.74
Low	866	1.02 (1.00, 1.05), 0.09	
High	867	1.03 (0.99, 1.07), 0.11	
HDL			0.59
Low	855	1.02 (0.99, 1.05), 0.23	
High	878	1.03 (1.00, 1.06), 0.04	
LDL			0.93
Low	865	1.02 (1.00, 1.05), 0.06	
High	868	1.03 (0.99, 1.07), 0.18	

*BUN* Blood urea nitrogen, *Cr* Creatinine, *HDL* High-density lipoprotein, *LDL* Low-density lipoprotein

Note 1: Above model adjusted for Age; Blood platelet; Albumin; Stroke severity; Triglyceride; LDL

Note 2:In each case, the model is not adjusted for the stratification variable

## Discussion

This study revealed that BUN/Cr is significantly associated with HT in a linear fashion in AIS patients, with an apparent inflection point demarcating the HT difference. These results support a revision in how we anticipate the prognosis for AIS patients. In addition, we found an interaction between BUN/Cr and DM and HT (*P* for interaction = 0.04). BUN/Cr had a positive correlation with HT (OR = 1.07; 95% CI: [1.02, 1.12]) in DM patients but no significant relationship with HT in patients without DM.

As mentioned before, an elevated BUN/Cr indicates serious medical conditions and poor prognosis in patients with acute kidney injury and acute heart failure [8, 12]. Previous studies have shown that impaired kidney function involves an excess risk of bleeding, which might contribute to platelet dysfunction coupled with abnormal platelet–blood vessel wall interaction [27]. Renal dysfunction has also been reported to be linked with small-vessel cerebrovascular disease and increased risk of haemorrhagic microangiopathy, which might be eventually attributable to cerebral haemorrhage [28–31]. Therefore, an elevated BUN/Cr might explain why the risk of HT increases in AIS patients. In addition, kidney impairment has been linked to inflammation as well as endothelial dysfunction. Microinflammation in kidney impairment might improve vascular activation and leukocyte infiltration in the etiopathogenesis of HT. Finally, bleeding in patients with kidney impairment might be due to a disturbance in the coagulation system [29]. On the basis of our data and these existing reports, we speculate that an elevated BUN/Cr increases the risk of HT probably by increasing the prevalence of small-vessel cerebrovascular disease. This should be confirmed directly by the biomarkers of small-vessel diseases.

It is essential to explore subgroup analyses for a scientific study [32]. In this study, we found that an elevated BUN/Cr has a positive correlation with HT in AIS patients with DM. About 30% of AIS patients have DM, and diabetics suffer the worst clinical outcomes from strokes [33, 34]; therefore, improving outcomes in the diabetic subgroup is of great importance. Studies have established a close link between HT and arteriosclerosis as well as microvascular impairment [35], which might be induced by persistent high blood glucose. Previous studies have also shown that DM/hyperglycaemia is linked to HT [36–41], indicating that DM might influence the process of haemorrhaging. With relative microvascular fragility [42], DM might account for earlier and greater cerebrovascular damage in HT patients. Therefore, our results could be interpreted as microvascular damage and arteriosclerosis being the major cause of HT [43–45] and BUN/Cr being an important parameter in appraising chronic vascular complications.

This study had several unique strengths. First, we discovered a nonlinear BUN/Cr–HT relationship, which might contribute to revealing the real association between BUN/Cr and HT. The GAM is good at handling nonlinear relationships because it can cope with nonparametric smoothing and configure a regression spline [26]. Second, although potential confounding factors in the study were unavoidable, we used strict statistical adjustment to minimize residual confounding. Third, modifier factor analysis took full advantage of the data. Also, subgroup analysis found a positive correlation of BUN/Cr with HT in DM patients. The study also had several potential concerns or limitations. First, this was an observational analysis and there were some differences in the baseline characteristics among the three patient groups. Second, the study was single-centre-based, so further studies are required in order to verify our extrapolation. The number of patients in each subgroup was relatively low, which might indicate undervaluation of the BUN/Cr–HT relationship. Third, symptomatic haemorrhage was not analysed independently. Four, data of a large infarct area, the degree of leukoaraiosis and a combination of biomarkers of small-vessel diseases were not analysed. Finally, the predictive effect of BUN/Cr on HT in different phases of AIS was not disclosed. Therefore, more high-quality studies are required in order to explore the different effects of BUN/Cr on HT in the early and late phases of AIS.

## Conclusion

The results of this study showed that BUN/Cr is an independent predictor for HT in AIS patients. BUN/Cr is positively correlated with HT when patients have DM. Further studies are required in order to elucidate the association between renal insufficiency and HT in terms of clinical outcomes after AIS, in addition to further confirmation of our findings in an independent study.

## Additional file

Additional file 1: **Figure S1**: Flow chat. **Table S1** The results of univariate analysis. (DOCX 231 kb)

## Abbreviations

AIS: Acute ischaemic stroke
BUN: Blood urea nitrogen
CI: Confidence interval
Cr: Creatinine
CT: Computed tomography
DM: Diabetes mellitus
eGFR: Estimated glomerular filtration rate
GAM: Generalized additive model
HDL: High-density lipoprotein
HT: Haemorrhagic transformation
LDL: Low-density lipoprotein
MRI: Magnetic resonance imaging
NIHSS: National Institutes of Health Stroke Scale
OR: Odds ratio
ORs: Odds ratios
