# Supplementary material for: Effects of the blood urea nitrogen to creatinine ratio on haemorrhagic transformation in AIS patients with diabetes mellitus
Source: BMC Neurol. 2019 Apr 13;19:63. doi: 10.1186/s12883-019-1290-x (PMC6463662; doi:10.1186/s12883-019-1290-x)
Supplement: Supplementary file 1 — Figure S1: Flow chat. Table S1 The results of univariate analysis. (DOCX 231 kb) [file 12883_2019_1290_MOESM1_ESM.docx]

**Effects of the blood urea nitrogen to creatinine ratio on haemorrhagic transformation in AIS patients with diabetes mellitus**

Linghui Deng, MD^1^*, Shi Qiu, MD^2,3^*, Changyi Wang, MD^1^*, Haiyang Bian, PhD^4^, Lu Wang, MD^1^, Yuxiao Li, MD^1^, Bo Wu, MD^1^ and Ming Liu, MD, PhD^1^

1 Center of Cerebrovascular Diseases, Department of Neurology, West China Hospital, Sichuan University, Chengdu, Sichuan, China

2 Department of Urology, Institute of Urology, West China Hospital, Sichuan University, Chengdu, Sichuan, China

3 Center of Biomedical big data，West China Hospital，Sichuan University, Chengdu, Sichuan, China

4 Department of Epidemiology and Biostatistics, School of Public Health, Peking

University, Beijing, China

*These authors contributed equally to this work

Correspondence to:

Ming Liu, MD, PhD, email: [wyplmh@hotmail.com](mailto:wyplmh@hotmail.com)

Bo Wu, MD, email: [dragonwb@126.com](mailto:dragonwb@126.com)

Additional file: Appendices 1-2

Appendix 1: Flow chat

Appendix 2: The results of univariate analysis

Figure S1: Flow chat


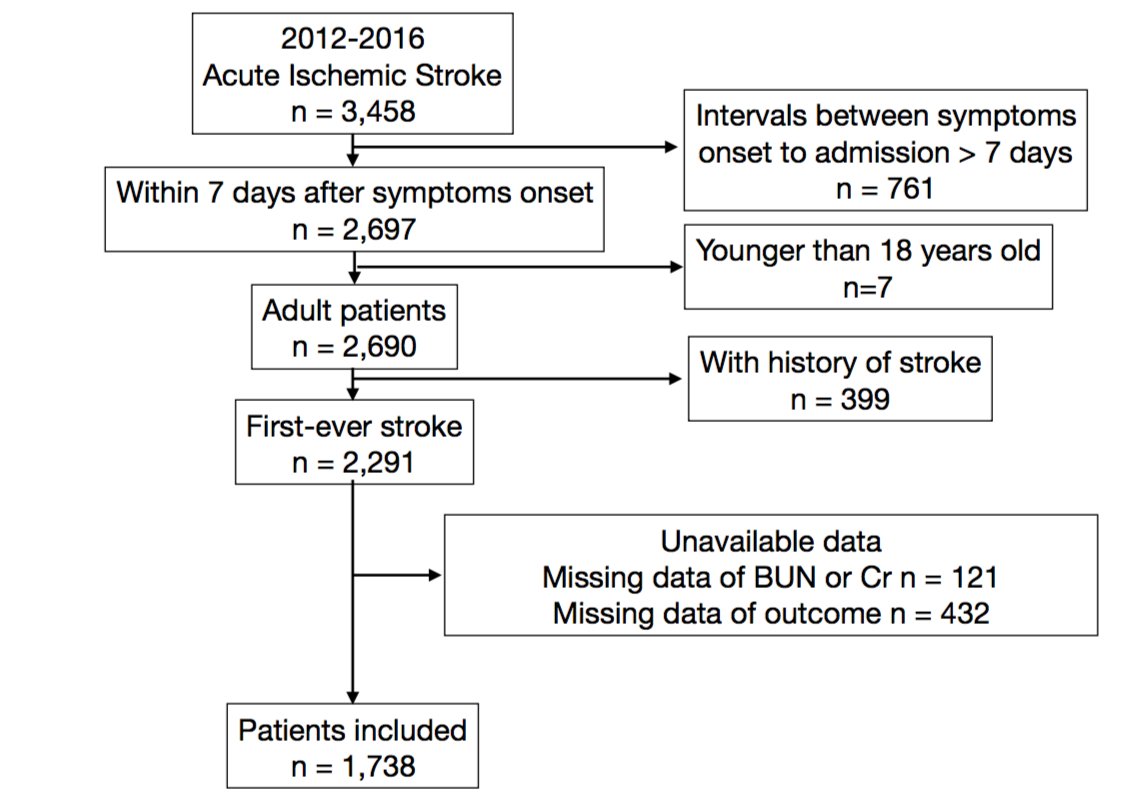


Table S1: The results of univariate analysis

|  | Statistics | Effect size （β） | P value |
| --- | --- | --- | --- |
| BUN/Cr | 18.72+6.87 | 1.03 (1.01, 1.05) | <0.01 |
| BUN/Cr (tertile) |  |  |  |
| T1 | 578 (33.26%) | Ref |  |
| T2 | 580 (33.37%) | 0.97 (0.61, 1.53) | 0.895 |
| T3 | 580 (33.37%) | 1.88 (1.25, 2.82) | <0.01 |
| Blood platelet count (*10^9^/L, mean ± sd) | 169.31+64.80 | 1.00 (0.99, 1.00) | <0.01 |
| Albumin (g/L, mean ± sd) | 40.76+4.56 | 0.96 (0.93, 0.99) | 0.01 |
| Triglyceride (mmol/L, mean ± sd) | 1.60+1.11 | 0.84 (0.69, 1.02) | 0.07 |
| Total cholesterol (mmol/L, mean ± sd) | 4.43+1.11 | 0.81 (0.69, 0.95) | 0.01 |
| HDL(mmol/L, mean ± sd) | 1.29+0.39 | 1.28 (0.85, 1.95) | 0.24 |
| LDL(mmol/L, mean ± sd) | 2.62+0.95 | 0.77 (0.64, 0.94) | 0.01 |
| Sex |  |  |  |
| female | 691 (39.76%) | Ref |  |
| male | 1047 (60.24%) | 0.76 (0.54, 1.06) | 0.10 |
| Age | 62.71+14.02 | 1.02 (1.01, 1.03) | <0.01 |
| Interval form symptom onset to admission (minutes) | 61.28+49.27 | 1.00 (0.99, 1.00) | 0.28 |
| Hypertension |  |  |  |
| none | 874 (50.29%) | Ref |  |
| yes | 864 (49.71%) | 0.85 (0.61, 1.19) | 0.34 |
| Diabete Mellitus |  |  |  |
| none | 1418 (81.59%) | Ref |  |
| yes | 320 (18.41%) | 1.39 (0.93, 2.07) | 0.11 |
| Hyperlipidemia |  |  |  |
| none | 1654 (95.17%) | Ref |  |
| yes | 84 (4.83%) | 0.52 (0.19, 1.43) | 0.20 |
| Atrial Fibrillation |  |  |  |
| none | 1604 (92.29%) | Ref |  |
| yes | 134 (7.71%) | 2.41 (1.48, 3.91) | <0.01 |
| Alcohol intake |  |  |  |
| none | 1293 (74.40%) | Ref |  |
| yes | 445 (25.60%) | 0.81 (0.54, 1.20) | 0.29 |
| Current smoking |  |  |  |
| none | 1133 (65.19%) | Ref |  |
| current | 605 (34.81%) | 0.79 (0.55, 1.13) | 0.20 |
| Stroke severity |  |  |  |
| baseline NIHSS score <15, n (%) | 1548 (89.07%) | Ref |  |
| baseline NIHSS score >=15, n (%) | 190 (10.93%) | 3.07 (2.04, 4.61) | <0.01 |

Ref: reference

BUN=blood urea nitrogen; Cr=creatinine; NIHSS=National Institutes of Health Stroke scale; HDL=high-density lipoprotein; LDL=low-density lipoprotein.
